# Guided self-help on the internet for turkish migrants with depression: the design of a randomized controlled trial

**DOI:** 10.1186/1745-6215-11-101

**Published:** 2010-11-04

**Authors:** Burçin Ünlü, Heleen Riper, Annemieke van Straten, Pim Cuijpers

**Affiliations:** 1 Department of Clinical Psychology, VU University, van der Boechorstraat 1, 1081 BT Amsterdam, the Netherlands; 2 Innovation Centre of Mental Health & Technology (I.COM), the Trimbos-institute, the Netherlands Institute of Mental Health and Addiction, P.O. Box 725, 3500 AS Utrecht, the Netherlands

## Abstract

**Background:**

The Turkish population living in the Netherlands has a high prevalence of psychological complaints and has a high threshold for seeking professional help for these problems. Seeking help through the Internet can overcome these barriers. This project aims to evaluate the effectiveness of a guided self-help problem-solving intervention for depressed Turkish migrants that is culturally adapted and web-based.

**Methods:**

This study is a randomized controlled trial with two arms: an experimental condition group and a wait list control group. The experimental condition obtains direct access to the guided web-based self-help intervention, which is based on Problem Solving Treatment (PST) and takes 6 weeks to complete. Turkish adults with mild to moderate depressive symptoms will be recruited from the general population and the participants can choose between a Turkish and a Dutch version. The primary outcome measure is the reduction of depressive symptoms, the secondary outcome measures are somatic symptoms, anxiety, acculturation, quality of life and satisfaction. Participants are assessed at baseline, post-test (6 weeks), and 4 months after baseline. Analysis will be conducted on the intention-to-treat sample.

**Discussion:**

This study evaluates the effectiveness of a guided problem-solving intervention for Turkish adults living in the Netherlands that is culturally adapted and web-based.

**Trial Registration:**

Nederlands Trial Register: NTR2303

## Background

Historically, the Netherlands has played an important role in Europe as a host country for immigrants. The immigration of guest workers became an important trend after World War II and changed the Netherlands into a multi-ethnic and multicultural society. Nowadays, almost one-fifth (19.9%) of the Dutch population was born outside the Netherlands (first generation migrants) or has one or both parents born outside the Netherlands (second generation migrants) [1]. Slightly more than half of these migrants (55%) come from non-western countries. The Turkish population is one of the main ethnic groups in Dutch society and constitutes 2.3% of the total population. About half of this group was born in Turkey (first generation), while the other half has parents who were born in Turkey (second generation). Migration is a complex process, which can have a immense impact on a migrant's life and mental health. It can improve the quality of life of migrants in the economic sense, but it can also involve complexities in the adjustment process, such as unemployment, minority status and tensions between generations [2].

One-third of the Turkish population living in the Netherlands has psychological problems such as depression and anxiety, which is a higher prevalence than normally found in general population studies [2]. The 1-month prevalence of depressive and/or anxiety disorders is highest among the Turkish migrant group (18.7%) in comparison with other ethnic groups (6.6% among Dutch and 9.8% among Moroccan) [3].Turkish women in particular currently have a strongly increased risk for developing depression compared to Dutch natives, and young Turkish women are at a high risk of attempting suicide [4].Despite the high risk of serious psychological problems among the Turkish population, they benefit to a much lesser extent from advances in evidence-based depression prevention services than the general population. Their mental health care service uptake is low and they often have a high threshold for seeking professional help for their mental health problems [5,6].

An innovative way to overcome the barriers referred to above is the Internet, which has low threshold acceptability, a high level of anonymity and offers flexibility in time and place. There is now convincing evidence that web-based interventions effectively reduce depressive symptoms and prevent depression [7-12] and they have been shown to be not only clinically effective, but also cost effective [13]. Little is known, however, about the effectiveness of these interventions in ethnic minority groups, although expectations are that these groups may also benefit from the interventions. Self-help through the Internet has the main advantage of a high level of anonymity and might overcome important cultural barriers. Since almost 80% [14] of the Turkish population in the Netherlands has internet access, it could be an ideal way of reaching and offering help to Turkish people with depressive symptoms.

One successful example of an evidence-based intervention is *Alles Onder Controle *(AOC). This is a web-based guided treatment defined as a standardized psychological intervention, which can be worked through independently by the clients themselves in their own homes. The clients receive weekly feedback from a trained coach by e-mail. *AOC *has a self-examination framework [15] and is based on problem-solving therapy, the core element being that clients learn to regain control over their problems and lives in a structured way. This method has been proven to be effective in several studies [16,17] and the statistical and clinical effectiveness of *AOC *in reducing symptoms of depression and anxiety among native Dutch have been shown in several studies [11,18]. It is still unknown, however, whether this intervention is also effective among ethnic groups, especially among the Turkish population, which is currently a high risk group for developing depression. No previous attempts have been made to adjust and examine *AOC *for Turkish migrants, although the limited studies available on depression interventions in other countries show that ethnic minorities can be effectively recruited and treated with prevention interventions [19-22].

In the present study, we will work with *AOC-TR*, the culturally adapted version based on the needs of the Turkish population that is available in Dutch and in Turkish. This online guided self-help intervention is intended to reduce depression complaints and the effectiveness of *AOC-TR *will be examined in a randomized controlled trial with adult Turkish people living in the Netherlands.

## Methods

### Study design

This study is a randomized controlled trial with two arms: an experimental group and a wait list control group. The experimental group obtains direct access to the guided web-based self-help intervention; the wait list control group receives access to the intervention after 4 months. The study protocol, information brochure and informed consent have been approved by the Medical Ethics Committee of the VU University.

### Inclusion and exclusion criteria

Turkish adults living in the Netherlands are eligible if they meet the following criteria: 1) age 18 years or older; 2) depressive symptoms (measured by the Center for Epidemiologic Depression Scale, CES-D score ≥ 16); 3) Turkish background (participant was born in Turkey or at least one of his/her parents was born in Turkey); 4) has access to a PC and the Internet and an e-mail address. Participants will be excluded if they are suicidal (according to the MINI-International Neuropsychiatric Interview).

### Recruitment

Recruitment will take place among the Turkish population in the Netherlands by means of advertisements in Dutch and Turkish national newspapers, magazines, community sites, and banners on health related websites for migrants. These advertisements contain a link to the research website with information about the study. Respondents who are interested can apply by sending an e-mail to the researcher. The information brochure and informed consent will be e-mailed together with the link for the screening.

### Randomization

After screening, participants will be randomized into one of the two conditions: the experimental group and wait list control group. The allocation schedule will be generated by an independent researcher using a computerized system. Those in the experimental condition will receive a username and password to log in to *AOC-TR*. Participants who are in the wait list condition will receive the same schedule of assessments online as the people in the experimental condition and receive a username and password to log in to the treatment after four months.

### Sample size

The sample size will be based on the expected difference of d = 0.45 on the primary outcome between the experimental and control groups. This expected difference is based on previous studies [10,11]. Based on a power (1-beta) of 0.80 in a two-tailed test, an alpha of 0.05, we need to start with 100 participants at baseline in each condition to show an effect-size of 0.45. The total sample size at baseline therefore, is determined as being 200 participants with depressive complaints.

### Interventions

#### Problem-Solving Treatment

The Dutch version of Alles Onder Controle has been adapted by:

- cultural sensitivity in the languages and presentation in relation to psychological problems

- use of culture-specific cases and problems that are recognizable for the target group concerned

- culture-specific examples of persons with similar problems

The intervention was translated into Turkish by a Turkish psychologist after adaptation and the translation was checked by two native speakers. Each participant is allowed to choose his/her language of preference.

The intervention consists of 5 sessions and takes 5 weeks in total. During that period the respondents indicate what they think is important in their lives, they make a list of their "problems and worries" and they categorize their problems into three groups: unimportant (not related to what they think is important in their lives), important and solvable (these problems are solved by a systematic problem-solving approach consisting of 6 steps), or important but unsolvable (having lost someone through death or having a chronic general medical disease for example; in the case of problems like these, they make a plan for how to live with them). The 6-step problem-solving procedure is the core of the intervention and people are stimulated to use this procedure during the course for several of their 'important and solvable' problems. The idea is that by mastering this technique people will regain mastery of their problems and ultimately their lives. At the end, participants will receive a certificate for completing the intervention.

#### Support

The participants are supported by trained coaches, whose feedback on the homework assignments done by the participants is given in brief weekly e-mails. The total amount of time spent on each participant is about 1.5 hours. It takes about 15 or 20 minutes per week per respondent to write these e-mails, which will be done by a bi-lingual psychologist at the VU University. The researcher (BU) will verify whether the coach has followed the treatment protocol sufficiently by reading a selection of the emails and by supervision.

### Assessments

All assessments will take place in the preferred language, either Dutch or Turkish. The primary outcome measure is the reduction of depression symptoms as measured by the CES-D, the secondary outcome measures are symptoms of anxiety, somatic symptoms, acculturation, quality of life and satisfaction.

The CES-D, one item on the Beck Depression Inventory-II (BDI-II) and Section C of the Mini-International Neuropsychiatric Interview (M.I.N.I) will be administered as screening measures. Suicide ideations and risk will be measured in two steps. Suicide ideations and risk will be measured in two steps. First, the suicide item on the BDI-II will be presented [23,24]. This instrument is validated among Dutch (e.g. [25,26]] and Turkish populations [27,28]. If the response is confirming, then the suicide risk will be measured with the suicidality section of the MINI [29,30]. The suicide section of the MINI consists of six items and quantifies respondents in four groups: no suicide risk, low suicide risk, moderate suicide risk and high suicide risk. Dutch [31] and Turkish [32] translations of the MINI will be used.

**Figure 1:**
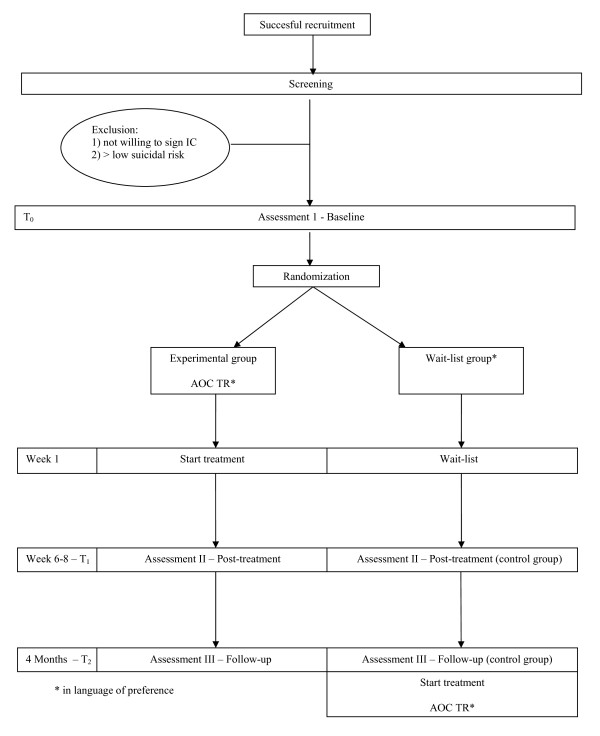
**Research procedure in flow diagram**.

Assessments will take place before randomization (T_0_), after completing the treatment (6-8 weeks, T_1_), and four months after baseline (T_2_). An overview of the procedures the participants will undergo in this study is given in Figure 1. See table 1 for an overview of all assessments at each point in time.

**Table 1:** Overview of instruments per measurement.

	Number of items	**T**_**0**_ (baseline)	**T**_**1**_ (6-8 weeks)	**T**_**2**_ (4 months)
1. Socio-demographic data	10	***x***		

2. Depressive symptoms (CES-D)	20	***x***	***x***	***x***

3. Anxiety (scale of HADS)	7	***x***	***x***	***x***

4. Somatic symptoms (scale of SCL-90)	12	***x***	***x***	***x***

5. Acculturation (LAS)	28	***x***	***x***	***x***

6. Quality of life (EuroQol 5D)	5	***x***	***x***	***x***

7. Satisfaction and track and trace	5		***x***	***x***

### Instruments

#### Depressive symptoms

Depressive symptoms will be measured using the Center for Epidemiologic Depression Scale (CES-D) [33]. It includes 20 self-rated items, each scored 0-3, and measures the severity of depression. The total score ranges from 0 (no feelings of depression) to 60 (severe symptoms of depression. The CES-D is available in Dutch and Turkish and both have been proven to have good psychometric properties in terms of validity and reliability [34,35]. The CES-D is also reliable and valid when presented digitally [36]. The optimal cut-off score varies in literature, but a score of 16 is usually regarded as indicating clinically relevant depressive symptoms [37].

#### Anxiety

The Anxiety scale of the Hospital Anxiety and Depression Scale (HADS) will be used to measure symptoms of anxiety [38]. The HADS consists of a depression scale and an anxiety scale with a total of 14 items. Each item can be scored with a 4-point Likert scale on a range of 0-3, where 0 refers to no anxiety and 3 to high anxiety. The total score range is 0-21. A score between 0 and 7 indicates no anxiety; a score between 8 and10 indicates possible anxiety; scores above 11 or12 are indicative of a clinical anxiety disorder. The HADS has been proven to be a valid and reliable instrument in various normal and clinical Dutch samples [39,40] and in Turkish samples [41].

#### Somatic symptoms

The Somatization (SOM) subscale on the Symptom Checklist-90-Revised (SCL-90-R) will be used for measuring somatic symptoms [42]. This is a five-point rating scale containing 12 items. Dutch and Turkish translations will be used for this study [43,44].

#### Acculturation

The Lowlands Acculturation Scale (LAS) will be used to measure the degree of acculturation [45,46]. It consists of 25 items that are rated on 6-point Likert-type scales with the extremes labelled as 'totally disagree' and 'totally agree'. The LAS can be divided into 5 subscales: Skills, Traditions, Social Integration, Values and Norms, and Feelings of Loss. The official Dutch and Turkish translations will be used, both of which have been validated [45].

#### Quality of life

The quality of life will be measured by the EuroQol Questionnaire (EQ5D) [47] in the official Dutch and Turkish translations, both of which have been validated [48-50]. This instrument consists of 5 items (mobility, self-care, usual activities, pain/discomfort, and anxiety/depression), each of which is rated as causing "no problems", "some problems", or "extreme problems". The score per item ranges from 0 (poor health) to 1 (perfect health).

#### Satisfaction and Track and Trace

A track and trace system will keep a record of the dates the participant logs on or finishes a lesson, and the number of e-mails sent to and received by the coach. This system will also ask the question "Was this lesson useful to you?" in Dutch and Turkish after each lesson and the answer can be given on a 5-point Likert scale.

### Statistical analysis

The study will be carried out in accordance with the CONSORT guidelines. All analyses will be based on the intention-to-treat sample and missing values will be imputed by means of regression analyses. A t-test will be used to compare the post-test mean scores (at T_1 _and T_0_) for the intervention group with the post-test mean scores for the control group. For comparison of the two means Cohen's *d *will be used as the between effect size. Cohen's *d *will be calculated as the difference between the post-test mean scores of the intervention and the control group divided by the pooled standard deviation. Effect sizes of 0.8 are assumed to be large; effect sizes of 0.5 are moderate; and effect sizes of 0.2 are assumed to be small [51].

## Discussion

A substantial part of the Dutch population has a Turkish background. They have a high prevalence of mental health disorders and have a high threshold for seeking professional help for these problems, but providing psychological help through the Internet may lower this threshold. This study evaluates the effectiveness of a guided self-help problem-solving intervention for depressed Turkish migrants that is culturally adapted and web-based. The strengths and limitations of the study can be summarized as follows:

Much is still not known about the effectiveness of internet interventions for depression in ethnic groups. By examining the effectiveness of a problem-solving treatment on the Internet for Turkish adults in the Netherlands, important information about psychological treatments in an ethnic minority group will be gathered. This will be highly relevant for clinicians and mental health services in improving the quality and the effectiveness of offering professional help.

A second strength of this study is that the intervention is available in two languages, which provides the flexibility of choosing the language of preference for receiving professional help. Turkish migrants who were previously unable to seek help because of the language barrier can now be treated. Moreover, by using the Internet to offer help, those who have a high threshold for seeking help can be reached and benefit from the treatment.

Another strength of this study is that the assessments will be carried out in two languages, while most other studies exclude non-Dutch speakers. All questionnaires used have been validated in both languages.

There are some limitations, however, that are worthwhile mentioning. First of all, we are unable for practical reasons to perform lengthy and costly diagnostic interviews, which means that we will not know how many participants in our study met the DSM criteria for depression. The results will, however, reflect the population whose symptoms are so distressing that they are willing to seek help. Secondly, the psychometric validities of the questionnaires used in this study have not yet been tested in an online environment.

## Abbreviations

AOC: Alles Onder Controle; AOC-TR: Alles Onder Controle TR (Adapted version); CES-D: Center for Epidemiologic Depression Scale; M.I.N.I.: Mini International Neuropsychiatric Interview; BDI-II: Beck Depression Inventory II; SOM: Somatization (subscale on the SCL-90-R); SCL-90-R: Symptom Checklist-90-Revised; LAS: Lowlands Acculturation Scale; EQ 5D: EuroQol Questionnaire 5D.

## Competing interests

The authors declare that they have no competing interests.

## Authors' contributions

All authors contributed to the design of this study. PC and AvS made the Internet-based PST intervention, AOC and BÜ adapted the intervention for the Turkish population. BÜ drafted the manuscript. All authors contributed to the further writing of the manuscript. All authors read and approved the final manuscript.
